# Obituary: Rupert Gerzer (1950–2025)

**DOI:** 10.1007/s00210-026-05243-7

**Published:** 2026-03-26

**Authors:** Klaus Aktories, Franz Hofmann, Jens Jordan

**Affiliations:** 1 Institute of Experimental and Clinical Pharmacology and Toxicology, Faculty of Medicine, University of Freiburg, Albertstr. 25, 79104 Freiburg, Germany; 2 Pharmakologisches Institut, Technische Universität, Biedersteiner Str. 29, D-80802 Munich, Germany; 3 Institute of Aerospace Medicine, German Aerospace Center (DLR), Linder Hoehe, D-51147 Cologne, Germany; 4 Medical Faculty, University of Cologne, Cologne, Germany

**Fig. 1:**
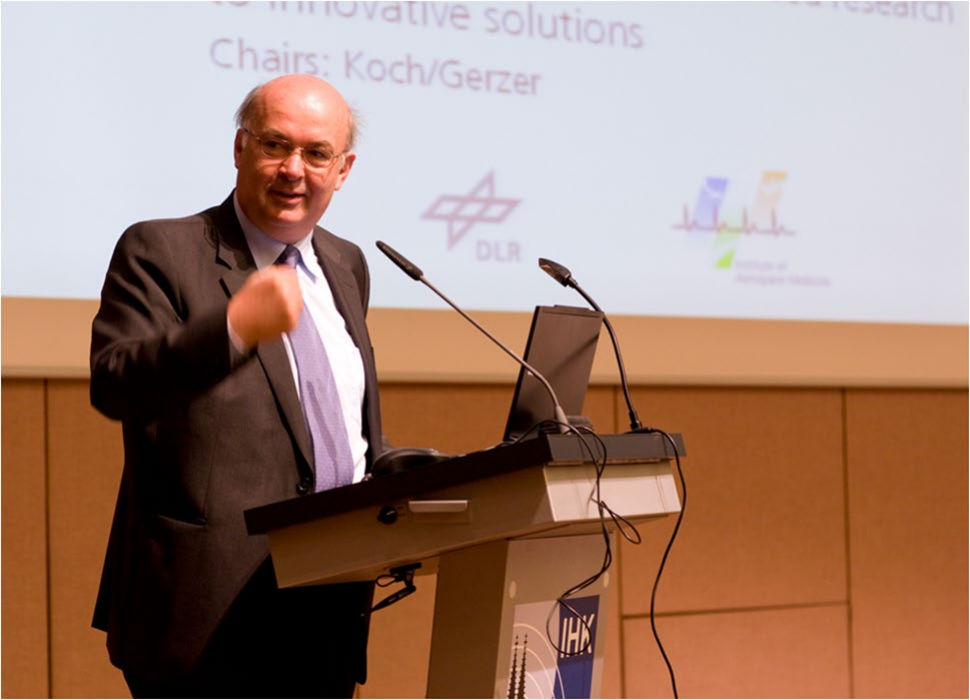
Prof. Dr. med. Rupert Gerzer (credit DLR)

Prof. Dr. med. Rupert Gerzer (Fig. 1) passed away on November 23, 2025, at the age of 75. With his passing, we have lost a physician and clinical pharmacologist whose pioneering work was fundamental to the development of space medicine in Germany and set international standards for the field.

Rupert Gerzer studied medicine in Munich and received his doctoral degree in 1977 with a dissertation on the role and regulation of cyclic nucleotides in rat tissue under the supervision of Prof. Klaus Loeschke. He then joined the laboratory of Günter Schultz in Heidelberg, where, together with Franz Hofmann and Eyke Böhme, he succeeded in purifying and characterizing soluble guanylyl cyclase (Drummer et al. 1990; Gerzer et al. 1981a). In the following years, his work focused on the production and physiological roles of guanylyl cyclases and the function of cyclic GMP. From 1981 to 1983, he worked as a visiting scientist in the laboratories of Joel G. Hardman and David L. Garbers at Vanderbilt University and the Howard Hughes Medical Institute in Nashville, USA. This stay further shaped his research and laid the foundation for his later contributions to guanylyl cyclase regulation and physiology (Gerzer et al. 1981b).

He returned to Munich from 1983 to 1988, where he completed his clinical specialization, achieved his habilitation in Pharmacology and Clinical Pharmacology (at the Medical Clinic – Innenstadt, Ludwig-Maximilian University of Munich), and obtained board certification in clinical pharmacology in 1990. Between 1988 and 1992, he held a Heisenberg C2 professorship in Munich. His research during this period was strongly influenced by the discovery of atrial natriuretic peptide (ANP) and its actions on particulate guanylyl cyclase (Gerzer et al. 1983). Several important papers by Rupert Gerzer and his colleagues elucidated the regulation, physiological functions, and pathophysiological roles of ANP in volume regulation and cardiovascular disease (Gerzer et al. 1985, 1994). In Munich, he also conducted pioneering studies on hormonal regulation during real or simulated spaceflight (Haufe et al. 1988), highlighting the early scientific interests that would later define his career.

From 1992 onward, Prof. Gerzer served as Director of the Institute of Aerospace Medicine at the German Aerospace Center (DLR) and as Professor (Chair) of Aviation Medicine at the Medical Faculty of RWTH Aachen University. His scientific work at the DLR produced internationally recognized findings in cardiovascular physiology, metabolic regulation, and human adaptation to microgravity. He established the DLR-Institute of Aerospace Medicine as a global leader in modern space medicine.

Together with his colleagues, he produced influential studies on body fluid and metabolism, demonstrating how microgravity alters fluid balance, electrolyte regulation, and energy requirements (Heer et al. 2000; Koch and Gerzer 2008; Müller et al. 1987). His research on the NO/cGMP signaling pathway clarified essential mechanisms of vascular regulation and contributed to the understanding of orthostatic intolerance after spaceflight. Under his leadership, the DLR-institute conducted pioneering studies simulating the effects of weightlessness on muscle, bone, metabolism, and the cardiovascular system.

At the DLR, Prof. Gerzer was instrumental in the conception, planning, and realization of the:envihab research facility in Cologne (Rakova et al. 2013). He championed the vision of creating an internationally unique, integrated research infrastructure for human studies that would enable long-duration, highly controlled investigations of human physiology, behavior, and performance. Through his scientific leadership and persistence,:envihab became a flagship facility for aeronautics and spaceflight-related human research worldwide, combining bed-rest studies, isolation experiments, and advanced metabolic and physiological measurements under one roof. This infrastructure has since served not only aerospace medicine but also terrestrial medical research, including studies on immobilization, aging, rehabilitation, and intensive care.

Beyond Germany, Prof. Gerzer played a decisive role in strengthening international aerospace life-sciences research. He was deeply involved in multinational research programs and maintained close scientific collaboration with the European Space Agency (ESA) and NASA, contributing German expertise to international missions and experiments on the Space Shuttle and the International Space Station. His ability to bridge disciplines and institutions helped position German space medicine as a trusted and influential partner in astronautical space exploration.

After his retirement from the DLR (2015), Prof. Dr. Rupert Gerzer accepted an appointment at Skoltech (the Skolkovo Institute of Science and Technology) in Moscow, an institution originally established in partnership and close collaboration with the Massachusetts Institute of Technology (MIT). At Skoltech, he served as Prorector (Provost), overseeing research and management of educational operations and also acting as the Skoltech representative for European relations.

Prof. Gerzer held numerous further honorary positions and received several distinguished awards throughout his career. He served as Chair of the University Council (Hochschulrat) of Bonn‑Rhein‑Sieg University of Applied Sciences (from 2007). He was President of the German Society for Aerospace Medicine (circa 1999–2001) and First Chair of the German Association for Travel Medicine. He was a member of the Scientific Advisory Board of the German Academy for Aviation and Travel Medicine and served on the Medical Advisory Board to the German Federal Ministry of Defence (from 2008 onward). He was a member of the Board of Trustees of the International Academy of Astronautics (IAA) (from 1999) and Editor‑in‑Chief of the journal Acta Astronautica (from 2008). His awards and honors include the Life Sciences Award of the International Academy of Astronautics (IAA) in 2003 and honorary membership in the International Association of Project Managers (IAPM), recognizing his contributions to aerospace medicine and international scientific cooperation.

With the death of Rupert Gerzer, we have lost an outstanding scientist who combined pharmacology and basic research with a visionary view of the future of medicine, as well as a highly esteemed colleague and friend.
